# Embodied Choice: How Action Influences Perceptual Decision Making

**DOI:** 10.1371/journal.pcbi.1004110

**Published:** 2015-04-07

**Authors:** Nathan F. Lepora, Giovanni Pezzulo

**Affiliations:** 1 Department of Engineering Mathematics, University of Bristol, Bristol, United Kingdom; 2 Bristol Robotics Laboratory (BRL), University of Bristol and University of the West of England, Bristol, United Kingdom; 3 Institute of Cognitive Sciences and Technologies, National Research Council, Rome, Italy; Brain and Spine Institute (ICM), FRANCE

## Abstract

Embodied Choice considers action performance as a proper part of the decision making process rather than merely as a means to report the decision. The central statement of embodied choice is the existence of bidirectional influences between action and decisions. This implies that for a decision expressed by an action, the action dynamics and its constraints (*e.g.* current trajectory and kinematics) influence the decision making process. Here we use a perceptual decision making task to compare three types of model: a serial decision-then-action model, a parallel decision-and-action model, and an embodied choice model where the action feeds back into the decision making. The embodied model incorporates two key mechanisms that together are lacking in the other models: action preparation and commitment. First, action preparation strategies alleviate delays in enacting a choice but also modify decision termination. Second, action dynamics change the prospects and create a commitment effect to the initially preferred choice. Our results show that these two mechanisms make embodied choice models better suited to combine decision and action appropriately to achieve suitably fast and accurate responses, as usually required in ecologically valid situations. Moreover, embodied choice models with these mechanisms give a better account of trajectory tracking experiments during decision making. In conclusion, the embodied choice framework offers a combined theory of decision and action that gives a clear case that embodied phenomena such as the dynamics of actions can have a causal influence on central cognition.

## Author Summary

The modern view of how we make perceptual decisions is of a process of accumulating sensory evidence until reaching a threshold level of certainty. However, this evidence accumulation model neglects the contribution of action and motor processes to the choice that is made. Recent novel studies that track the changing dynamics of actions during perceptual decisions are increasingly revealing the contribution of the actions we make to our perceptual choices. Thus, the action dynamics of our bodies causally influences our central cognition, which is a core assumption of embodied theories of mind. This paper presents an explanation of how actions, encompassing behavioral strategies such as preparation and commitment, can bias decision making processes in ways that optimize the ecological choices of animals behaving in natural environments. It thus combines two disconnected research streams, decision-making and action control in a manner consistent with theoretical and psychological arguments for embodied cognition.

## Introduction

According to a widely held view, perceptual decision making is a competitive process based on the accumulation of sensory evidence up to a threshold. This idea has been formalized using various methods, which include signal detection theory, neural race models, attractor dynamics and Bayesian inference [1–8]. However, although this diffusion-to-bound framework has received strong support from the neuroscience literature [9], it has been limited by only addressing decisions in isolation without considering if and how decision and action systems interact.

In this article, we propose a framework of *Embodied Choice*, in which action performance is considered as a proper part of the decision making process. To make comparison with previous work on decision making, we discuss three different ways to link decisions to action: the serial model, the parallel model, and the embodied choice model. We describe their theoretical underpinning first, before successively analyzing and comparing them from a computational perspective. The results of this comparison show that the serial model has a poorer speed-accuracy tradeoff than the parallel model; however, the parallel model is limited in that it cannot trade accuracy for speed to achieve the quickest decision times, which requires the full embodied choice model.

We first clarify the differences between these three types of decision making model in the remainder of the introduction, before giving results on how these models compare on a simple perceptual choice task involving action on synthetic data, and then give empirical support for embodied choice models from motion tracking experiments during decision making. Finally, in the discussion, we discuss the broader implications of an embodied choice framework and how it relates to recent developments in the neuroscience and psychology of decision making.

### Serial models: segregating decision and action

**Fig 1:**
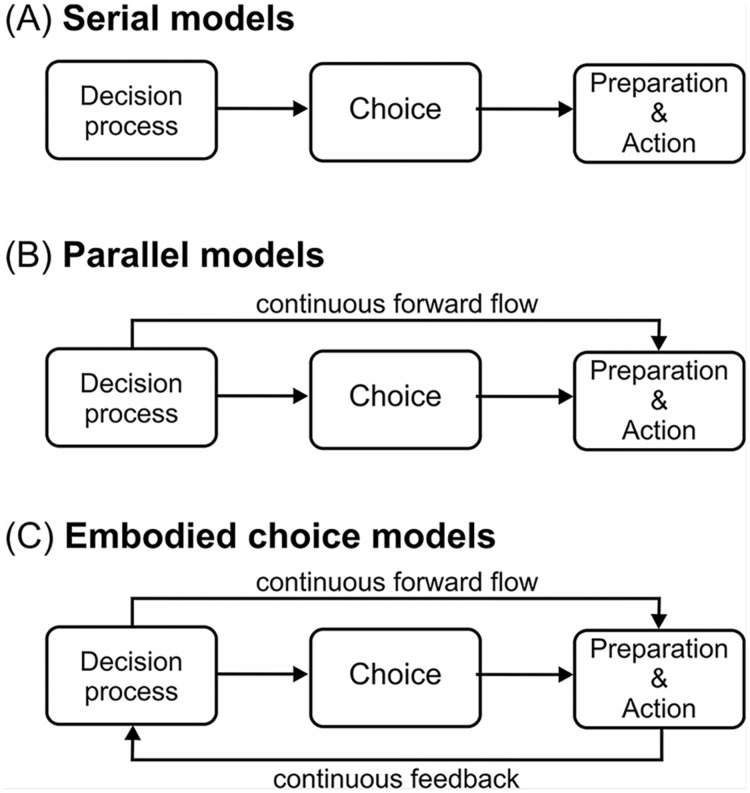
Schematic illustration of alternative ways to link decision and action systems: (A) serial models, (B) parallel models, and (C) embodied choice models. Note that here decision, choice, and action are shown as separate and modular systems for illustrative purposes only.

Most studies using the diffusion-to-bound framework implicitly assume that decision making is a *serial process* (although this is not a necessary feature of the diffusion-to-bound framework—see later). According to serial models (Fig. 1 (A)), decision and action are neurally/computationally segregated and arranged in a pipeline [10]: the decision is made first and then the action (*e.g.* pressing a button to report the choice) is executed at the time of decision completion; the costs of action are not considered.

### Parallel decision and action: the continuous flow model

That response regions in the brain show effects of decision signals before committing to the final outcome was known 25 years ago; for example, an EMG study found activation of the incorrect response hand in a flanker task, even on trials where subjects eventually gave the correct response [11]. Successive EEG and neurophysiological studies of decision-making consistently found a *covert* preparation of multiple motor plans in parallel, providing a strong support for parallel views of human information processing [12–15].

More recently, several studies tracking response dynamics (*e.g.* mouse movements of subjects who must click buttons to report choices) showed a similar effect for the *overt* phases of action. A consistent finding across several paradigms tracking mouse movements, from simple perceptual categorizations to more complex lexical and numerical decisions, is that overt action does not simply start when decision is complete. Instead, the action begins early in the decision process and is continuously revised, with action dynamics (*e.g.* mouse trajectories, acceleration peaks) reflecting the choice uncertainty [16–22].

This evidence has lead to the development of models in which decision and action are deployed in *parallel*. For example, in the *continuous flow model* [14], the partial results of the ongoing decision computations are continuously transferred to the action component (see Fig. 1 (B)). In this way action can start before completion of the decision and be revised during the decision process. See Table 1 for some differences between serial and parallel models of decision making.

**Table 1:** Differences between serial and parallel models of decision-making.

**Serial models**	**Parallel models**
Subjects accumulate evidence until they make a final decision (reach a bound) then act	Subjects start moving before they make a final decision and can revise the initial choice (change mind) successively
Decision is completed before starting the action	Decision is not completed before starting the action
Uncertainty in the decision is reflected in errors and response time	Uncertainty in the decision is also reflected in the movement trajectories

Note that the continuous flow model was initially developed to explain covert and not overt response competition. According to a recent proposal the continuous flow model can explain also overt revisions of actions during decision making and in particular so-called “changes of mind”, or the fact that an initially preferred choice is changed during the course of the decision making [23]. In this model, a change of mind happens when the drift-diffusion process first reaches a threshold that triggers action initiation, but then later (due to accumulated evidence not initially considered) passes an opposition threshold that triggers execution of an initially non-preferred action.

Another way the continuous flow model can explain overt response competition is to implement an *action preparation* strategy: the partial results of the competition are used to approach the more likely option (*e.g.* the more likely response button to be clicked with a mouse) or to move in between the alternatives if these are uncertain [24]. This can be realized, for example, by allowing the action system to generate two simultaneous motor commands and to average them over time with changing weights [25], producing a ‘continuous competition’ between response alternatives [20]. This strategy can explain why, during perceptual choices, participants’ movements (*e.g.* mouse trajectories) start almost immediately and can be attracted to the unselected alternative.

### Embodied choice

Both of the aforementioned decision making models consider decisions as independent of action and its dynamics. However, in ecologically valid scenarios, the situated aspects of choice (*e.g.* the action dynamics and the achievability of the action alternatives) should be worth considering as part of the decision making process. Ecological psychologists have long recognized that agents do not passively receive sensory stimuli, but contribute with their actions to shaping the sensory flux [26–28]. Not only sensations determine (choice and) action but actions determine the next sensations in a continuous perception-action loop. Within this loop, several aspects of action and its dynamics can influence the choice, as we now discuss.

First, in ecologically valid scenarios, actions are not effortless but have a cost. For example, reaching a far object is energetically more costly than reaching a close object, and takes more time. Hence, living organisms typically trade off the benefits of an outcome from the cost of obtaining it. In accordance, the biomechanical costs of movement and target distance influence choice [29–31], motivating recent computational studies that begin to incorporate action costs in the decision process [24, 32]. In principle, even serial and parallel models can incorporate action cost in the decision process; for example, they can trade off the benefits of pressing a button and the required effort. However, these models can only include costs that are known *a priori* and not those that emerge (or change) due to action dynamics, because they do not consider the feedback process from action to decision. In ecologically valid situations, costs cannot be completely specified *a priori* because they change in an action-dependent way. For example, the biomechanical costs of changing a decision (*e.g.* the choice of whether a lion should continue pursuing a gazelle or give up) will vary depending on the relative trajectory and the distance from the target.

This leads us to a second point: actions are not instantaneous but require time to execute. Imagine, for example, a lion given the choice between capturing two gazelles: if the lion waits until its decision is complete, it risks missing an opportunity because one or both gazelles may run away. The lion faces a decision problem that is not stable but dynamic. In dynamic, real-world environments, costs and benefits cannot be completely specified in advance but are defined by various situated factors such as the relative distance between the lion and the gazelles, which change over time as a function of the geometry of the environment (*e.g.* a gazelle jumping over an obstacle can follow a new escape path) and the decision maker’s actions (*e.g.* if the lion approaches one gazelle the other can escape) [33]. It seems that the brain solves this problem by preparing multiple actions in parallel and selecting between them through biased competition in the sensorimotor system [34, 35]. Furthermore, choice experiments conducted to assess the relative contribution of perceptual evaluation and motor execution to response time (using a ‘compelled response’ paradigm where participants need to initiate a saccade towards a target before the stimulus signalling the correct target is fully revealed) provide strong evidence in favor of the idea that action plans can be prepared, and even launched, prior to the completion of the decision process [36]. The continuous flow model can partially capture these processes by allowing the preparation of actions through the influence of the ongoing decision; however, as we noted before, it has principally focused on the covert components of preparation (*e.g.* allocation of neuronal resources) rather than also its overt components (*e.g.* initiation of movements towards or between alternative goal locations).

Recent computational studies have suggested a *continuous preparation* of action influenced by the current state of the decision process, and explored the trade-offs between alternative preparation strategies (*e.g.* approach one gazelle early with the risk of later undoing that action, or moving between them to keep all options open) [24], see also [37]. What these computational studies suggest is that action dynamics in all their aspects (*i.e.* both their covert planning and their overt execution) have a *backwards influence on the decision process* by changing the prospects (the value and costs of the action alternatives). For example, when the lion starts tracking one of the gazelles, undoing that action can be too costly and thus the overall benefit of continuing to track the same gazelle increases. This produces a *commitment* effect to the initial choice that reflects both the situated nature of the choice and the cognitive effort required for changing mind at later stages of the decision [38].

Accounting for all these aspects of ecological decision making requires a re-conceptualization of the relation between decision and action. To achieve this aim, we introduce an *Embodied Choice framework* in which action and its dynamics are considered an integral part of the decision making process, rather than merely a way to report an already made choice. The brain considers both the decision part (*i.e.* making the right choice) and the action part (*i.e.* minimizing movement costs) simultaneously. A consequence of this view is that actions and their constraints influence decisions. Fig. 1 (C) illustrates this aspect by including a feedback loop from the action to decision systems that is missing from both serial and parallel models. Table 2 further clarifies the differences between embodied choice and continuous flow models of decision making.

**Table 2:** Differences between parallel (continuous flow) and embodied choice models of decision-making.

**Parallel (continuous flow) models**	**Embodied choice models**
Action and its dynamics do not influence decision making optimization	Action and its dynamics directly influence decision making optimization
The partial results of the choice computation flow forward to the action system	In addition, action dynamics influence (e.g. provide evidence for) the decision making process
The forward influence from decision to action system permits changes of mind and motor preparation	The backward influence from action to decision produces commitment
The deadline for a decision is a threshold (or bound) within the decision system	The natural deadline for a decision is the completion of the situated action

Embodied choice can thus be considered a general framework that can be implemented in different ways. In this article we propose a computational characterization of *Embodied Choice* and compare it with alternative proposals made in the literature. We keep the model minimalistic (*e.g.* by using simplified movement dynamics) to better highlight the essential characteristics of embodied choice in comparison with alternative models.

## Results

Serial, parallel and embodied choice models make different assumptions on how decision and action systems interact. Here we incorporate these assumptions in four computational models and test them in a simulation of a simple perceptual choice task involving action. This test aids understanding of the differences between the alternative theoretical models while elucidating their predictions.

**Fig 2:**
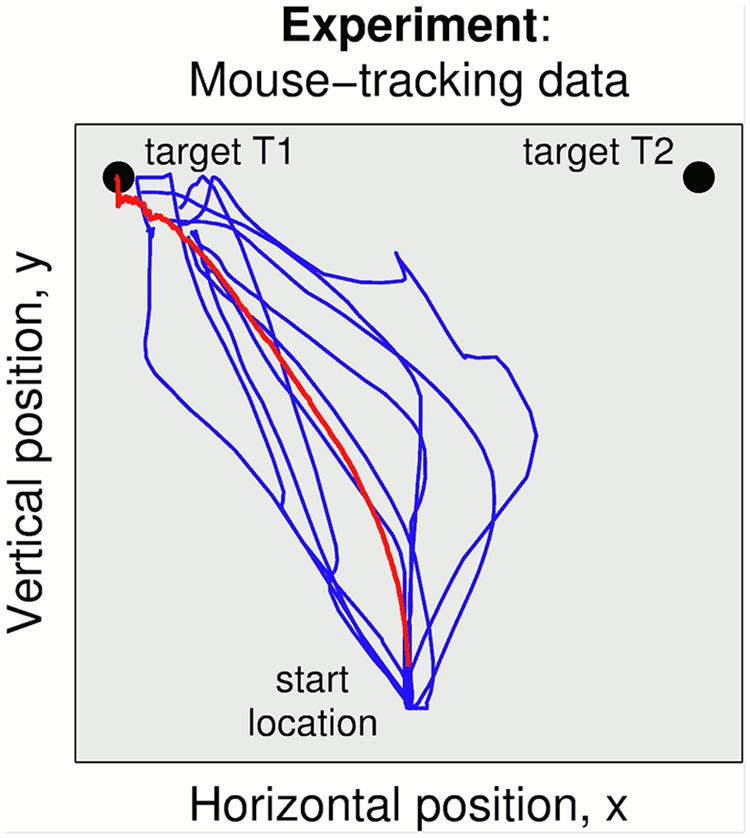
Mouse-tracking data. An action is made to either target T1 or target T2 to indicate the decision. This action has a trajectory beginning at a start position equidistant from both targets. The red line indicated the mean trajectory. Experiment: 10 trajectories randomly chosen from the data in ref. [16].

All simulations represent a two-alternative forced choice task (2AFC) in which an action must be made to one of two targets to indicate the decision (Fig. 2). For concreteness, we consider a task based around a computer mouse experiment in which the subject moves a cursor onto a target while making a perceptual choice (*e.g.* a lexical decision [16, 39]). However, we emphasize that this model is considered here as more broadly representative of any decision making task using action to make the choice.

These results are separated into two studies. In the first study, we simulate the decision trajectories for four computational models with increasingly sophisticated interaction between the decision making and action components. Model 1 serially initiates action after decision completion; Model 2 is parallel by allowing changes of mind in the decision system after the action initiates; Model 3 has action preparation operating in parallel with decision system, with also some aspects of embodiment by using action completion for decision completion; and Model 4 is fully embodied with a feedback loop from the action to decision systems, encompassing both action preparation and commitment. The trajectories from these four models are then compared with motion tracking experiments during decision making to provide empirical support for the models. Then in Study 2 we compute the speed-accuracy tradeoff curves resulting from these four models, and compare their performance to assess the overall effectiveness of serial, parallel and embodied choice models.

### Study 1: Decision trajectories during embodied choice

#### Experimental task: mouse-tracking experiment

Continuous measures of processing are more informative about the dynamics of choice than response time experiments. Thus, we model an experimental task in which participants performed the decision by moving the mouse to indicate their response. Experimental data was collected using a MouseTracker apparatus [17] during a visual-lexical decision task to observe the graded effects of competing items attracting the trajectory of the mouse [16]. By tracking continuous reaching movements, the technique enables study of the dynamics of choice between multiple competing hypotheses and can reveal graded processing and uncertainty throughout the response. Some example of mouse-tracking trajectories are shown in Fig. 2.

In our model of this task, we consider a square arena within which the mouse pointer follows a two-dimensional trajectory (*x*(*t*), *y*(*t*)). The choice is indicated when the mouse pointer reaches one of two targets: T1 at position (*x*_1_, *y*_1_) and T2 at (*x*_2_, *y*_2_).

#### Modeling mouse trajectories: drift-diffusion model with action focus

All computational models that we compare below are built on the drift-diffusion model [1], which is a model of the cognitive processes involved in making simple two-choice decisions. Decisions are made by a noisy process that accumulates information *z*(*t*) over time *t* (Fig. 4) from a starting point (assumed at *z*(0) = 0) towards one of two decision boundaries that initiate a response (assumed equal and opposite at *z* = ±*b*). Mathematically, the information accumulation can be defined as

$$\begin{array}{c} z(t+\Delta t)=z(t)+\Delta z, \end{array}$$ (1)

where Δz is the increment of sensory information over a time increment Δ*t*. The rate of accumulation of information is called the drift rate, and is determined by the quality of the information extracted from the stimulus in perceptual tasks and the quality of match between the test item and memory in memory and lexical decision tasks. Within-trial variability (noise) in the accumulation of information from the starting point toward the boundaries results in processes with the same mean drift rate terminating at different times (giving response time distributions) and sometimes at the wrong boundary (producing errors). Speed-accuracy trade-offs are modeled by changing the distance between the decision boundaries: wider boundaries (larger *b*) require more information to make a decision, leading to more accurate but slower responses. For further details of the model, see Ratcliff and McKoon [40].

**Fig 3:**
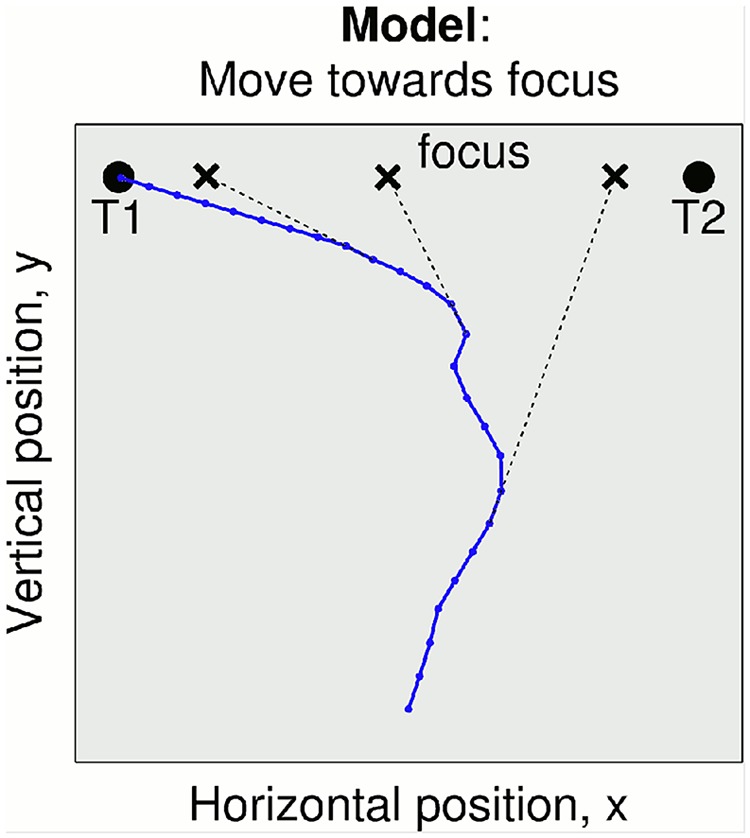
Simulated 2AFC task with action dynamics (trajectory from Model 3). For simplicity, movement along the trajectory is at constant velocity. The trajectory depends on the interaction between the decision and action systems, driven by information for the alternatives. An action focus is used to link the decision and action systems, which here drifts from target T2 to T1 with the accumulated information for each alternative.

To formalize tasks using continuous measure of performance (like the MouseTracker) we supplement the drift-diffusion model with an action focus: here a point towards which the agent moves based on the current state of the decision process and its current position (Fig. 3). Mathematically, we represent the action focus as a point (*x*(*z*), *y*(*z*))_focus_ that is a function of the accumulated information *z*(*t*). At each instance of time, the action is a move (Δ*x*, Δ*y*) from the present location towards the action focus

$$\begin{array}{c} \left(\Delta x\left(t\right),\Delta y\left(t\right)\right)=v\Delta t\frac{{\left(x\left(z\right),y\left(z\right)\right)}_{\mathrm{focus}}-\left(x\left(t\right),y\left(t\right)\right)}{{|\left(x\left(z\right),y\left(z\right)\right)}_{\mathrm{focus}}-\left(x\left(t\right),y\left(t\right)\right)|}, \end{array}$$ (2)

which for simplicity we assume is at constant speed *v* taken at discrete time-steps of Δ*t*.

The action focus provides a way in which the decision and action systems can interact, resulting in a continuum of models from fully non-embodied (*i.e.* where decision and action are two serial stages and only the former can influence the latter) to fully embodied (*i.e.* where decision and action systems interact bidirectionally and continuously). Below we consider four models in order of increasing sophistication and adherence to the characteristics of embodied models as defined in Table 2.

#### Model 1: Action initiation after decision completion

The simplest way to augment the drift diffusion model with an action system is to consider a serial model with two stages: first the decision is made at time *t*_dec_ (when the decision variable first passes a boundary), which then initiates the appropriate movement to enact that decision. For simplicity, we assume a basic action model with constant movement speed, with then the time-optimal action to move in a straight line towards the chosen target. This action model can be represented by having an action focus that is coincident on the chosen target for times *t* ≥ *t*_dec_

$$\begin{array}{c} {\left(x\left(z\right),y\left(z\right)\right)}_{\mathrm{focus}}=\left\{\begin{array}{cc} (x_{1},y_{1}), & z(t_{\mathrm{dec}})\geq b\\ (x_{2},y_{2}), & z(t_{\mathrm{dec}})\leq -b. \end{array}\right. \end{array}$$ (3)

**Fig 4:**
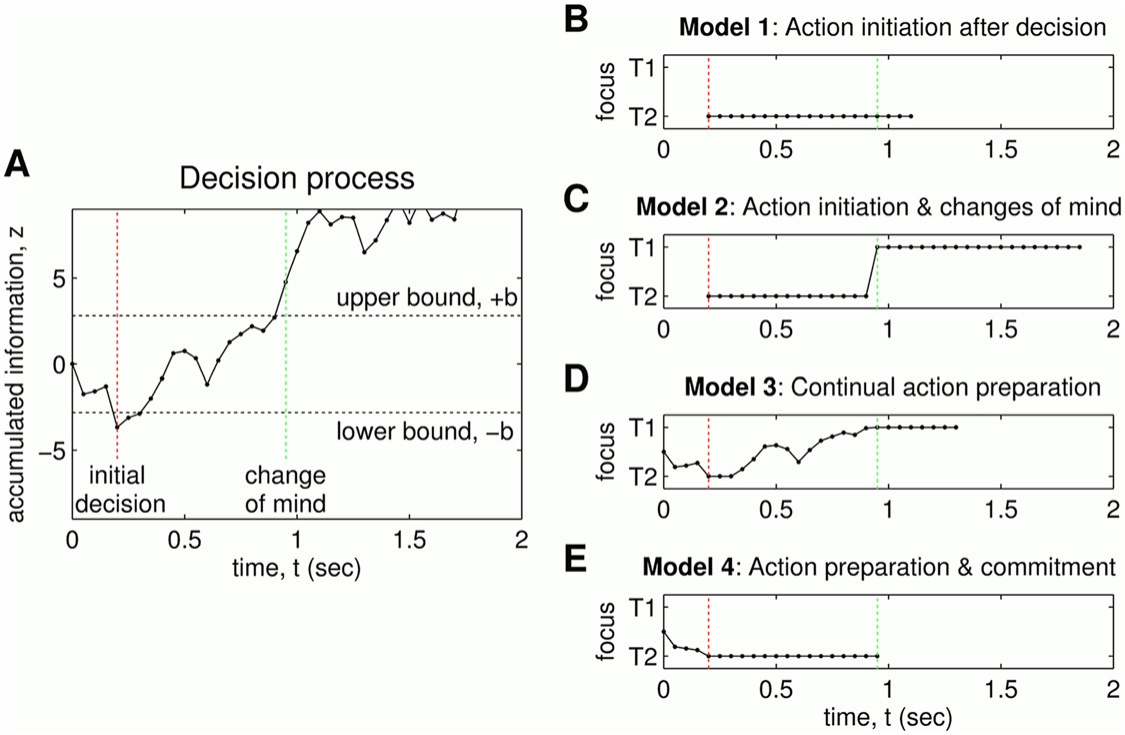
Accumulated information and motor response during the decision process. The top plot shows the accumulated information *z*(*t*) plotted against time, with decision bounds also shown and the times of crossing (dashed lines). The bottom plots show the trajectory of the focus on the line between the two targets. Model 1 (action initiation after decision) and Model 2 (action initiation and changes of mind) begin when the information first crosses threshold, with Model 2 changing its mind. Model 3 (action preparation) and Model 4 (preparation and commitment) begin at time zero, with the focus moving continually throughout the decision process.

Prior to the decision time, there is no choice and the agent cannot move (equivalently, the focus is at the start location). An example time-history of the action focus is shown in Fig. 4 as information is accumulated for the two choices during a decision process.

#### Model 2: Action initiation and changes of mind

Next, we consider a model that also initiates movement after passing a decision boundary, as in Model 1, but updates the decision variable with new sensory information during the movement. Consequently, if the opposing decision barrier is passed before the trajectory reaches a target, then the action is revised to move towards the other target. The possibility to revise an initial decision is a characteristic of parallel models, where the decision system does not stop functioning after a deliberation. Note that this model does not need a ‘continuous flow’ of information from decision to action systems [14]; it is sufficient that the decision system instructs the action system when the action is revised. This Model 2 is similar to a model of ‘changes of mind’ by Resulaj *et al* [23], although that model used distinct decision- and change-boundaries whereas the present model just has a change boundary with action completion triggering the final decision, to consistently represent the situated demands of the choice. Such changes of mind can improve decision accuracy by allowing choices to be revised after receiving initially misleading sensory data, but are penalized by longer response times due to sub-optimal ‘kinked’ trajectories to reach a target (*e.g.* that of Fig. 3). This action model can be represented by having an action focus that is coincident on the currently chosen target

$$\begin{array}{c} {\left(x\left(z\right),y\left(z\right)\right)}_{\mathrm{focus}}=\left\{\begin{array}{cc} (x_{1},y_{1}), & z(t)\geq b\\ (x_{2},y_{2}), & z(t)\leq -b. \end{array}\right. \end{array}$$ (4)

Similarly to Model 1, if the accumulated information has not passed threshold, then there is no choice and the agent cannot move. Again, an example time-history of the action focus is shown in Fig. 4.

#### Model 3: Action preparation

Next, we consider a parallel model of preparatory motor response from the start of the decision process, rather than waiting for a decision to trigger action initiation. In general, proactive action preparation consists of readying oneself so that successive actions can be executed better [24]: in the two-target 2AFC task considered here, we consider a preparatory move towards an action focus between the two targets, to approach the most likely target prior to accumulating sufficient information to make a decision. Note that calculating the action focus as a function of the ongoing decision (*e.g.* as a function of the target likelihood) requires a ‘continuous flow’ of information from decision to action systems. Mathematically, we define the focus as collinear with the two targets with distance from each in the proportion ∣*b* + *z*∣ : ∣*b* − *z*∣ for −*b* ≤ *z* ≤ *b*:

$$\begin{array}{c} {\left(x\left(z\right),y\left(z\right)\right)}_{\mathrm{focus}}=\left\{\begin{array}{cc} (x_{1},y_{1}), & z(t)\geq b\\ \frac{\left|b-z\right|}{2b}\left(x_{1},y_{1}\right)+\frac{\left|b+z\right|}{2b}\left(x_{2},y_{2}\right), & -b\leq z(t)\leq b\\ (x_{2},y_{2}), & z(t)\leq -b. \end{array}\right. \end{array}$$ (5)

This range is bounded such that the focus is coincident with a target if the decision bound is passed. Hence, the decision bound no longer defines decision termination directly, but rather the choice follows indirectly from action completion (upon reaching a target). Models 2 and 3 incorporate one element of embodiment because they use action completion as a deadline for the decision. In other words, in these models the decision is terminated when reaching a target, in contrast to the non-embodied method of having the decision complete upon reaching a decision bound. However, they are not a fully embodied models (as defined in Table 2) because the interaction between the decision and action systems is not bidirectional.

#### Model 4: Action preparation and commitment

Our final model implements embodied choice with both a preparatory motor response and commitment to an action when sufficiently engaged in moving towards a target. This model is built on the same mechanism as Model 3, with a preparatory move towards an action focus between the two targets, but also has commitment: movement towards a target biases the focus to be closer to that target. The dependence of the action focus upon both the accumulated information and the current movement and location is characteristic of embodied choice models, because such models require a feedback loop from action to decision systems. Mathematically, we implement commitment by including a position-dependent term in the accumulated information

$$z_{\text{com}}\left(t\right)=z\left(t\right)+g\frac{d_{1}-d_{2}}{d_{1}+d_{2}},\;d_{1}=\;|\left(x,y\right)-\left(x_{1}y_{1}\right)|,d_{2}=|\left(x,y\right)-\left(x_{2},y_{2}\right)|,$$ (6)

with *d*_1_, *d*_2_ the distances to the two targets and *g* is a gain that determines the degree of commitment (here set to *g* = 4*b*). The model is then fully embodied, because there is direct feedback from the action system (represented by the location (*x*, *y*)) into the decision system as a component of the evidence (see Fig. 1C). In this case, this creates a *commitment effect*: once you start moving towards a target, it is more difficult to change your mind because the sensory information *z* must outweigh the commitment from having initiated an action.

#### Empirical support for embodied models

The embodied choice framework assumes that choice is biased by the constraints of action, which are directly considered in the decision process. Not only is this true at the beginning of a choice but also during the unfolding of the action (*e.g.* while a subject is moving the mouse to press a button). The framework thus makes a number of predictions, some of which are supported by available data while others require future investigation. Here we focus on studies based on tracking mouse movements (see [16, 18, 21]).

**Fig 5:**
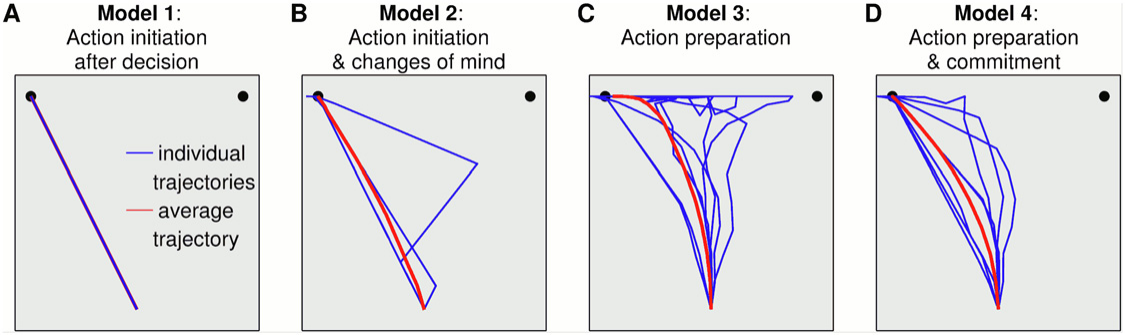
Individual and average trajectories. Models 1–4: 10 trajectories randomly taken from the analysis for Figs 3, 4. Trajectories were selected to complete at target 1 to the left. The decision bound *b* was chosen for Models 1–3 to have a mean response time of 1.5 sec; whereas (1 sec for model 4 to be in range for this model). The average trajectory (red plot) in each case is taken over 150 trajectories, and computed parallel to the *x*-axis.

In particular, the experimental results from mouse tracking studies [16] resemble those from the parallel Model 3 and embodied choice Model 4, as can be seen from comparing their individual trajectories (Fig. 5, Models 3 and 4) with experimental data (Fig. 2). Notably, while the trajectories of Model 2 head towards one choice then undergo a distinct change of mind to the other, the trajectories of Models 3 and 4 can occasionally produce discrete revisions but also graded shifts between choices (Fig. 5) that are interpreted as reflecting a process of graded, continuous competition among the alternatives [41]. Both patterns are observed in the human studies [16, 18, 21, 42]. Interestingly, both discrete and gradual revision processes have been reported in the same study and even in the same experimental condition, pointing to the necessity of a model that accounts for both [39].

These differences in changes of mind can be quantified by determining the area between the trajectories and a line running down from the chosen target (always shown to the left in Fig. 5), which we evaluate over all trajectories before taking the mean. The larger this average area, the more the trajectories deviate from heading directly towards the final choice. The minimum area is for Model 1 which always heads directly towards the chosen target (mean area: 0.75), followed by Model 2 (mean area: 0.91), then Model 4 (mean area: 1.06) and the largest is Model 3 (mean area 1.25). These values compare with a mean area of 1.03 for the Experimental Data, which is closest to that of Model 4 (consistent with a visual comparison of Fig. 5). Note that these values do depend on the noise levels *σ* assumed in the model, as is apparent in the ‘curvature’ of mouse movements discussed below. The sharp kink with the change of mind in Model 2 does not occur in human studies, although the modeled trajectories can be smoothed with minor model amendments such as limiting the angular velocity; such smoothing would also occur naturally with a more realistic model of the action kinematics [43]. However, the sharp kink does serve to illustrate that there is a distinct change of mind in this model, whereas human studies show both distinct changes of mind and more gradual shifts from one choice to the other as in Models 3 and 4.

Another key empirical finding is that in all the aforementioned mouse-tracking experiments (and others) the initial movements do not necessary target one of the two choice alternatives but often lie between them. Fig. 5 shows individual trajectories from the sample task introduced earlier: while in the serial Models 1 and 2 the initial parts of the trajectories already point towards one of the two buttons, in Models 3 and 4 this is not necessarily the case, as observed empirically in the Experimental Data and in a variety of studies [16, 18, 21, 42]. This finding can be quantified with the average trajectories (Fig. 5; red curves), which are initially almost vertical for the Experimental Data (initial angle from vertical: 6 deg) and Models 3 and 4 (angle: 2 deg, 7 deg), but off-vertical for Models 1 and 2 (angle: 34 deg, 21 deg).

It is interesting that embodied choice Model 4 (action preparation and commitment) more closely resembles the Experimental Data than the parallel Model 3 (action preparation only); for example, the initial trajectories have a similar angular bias towards the target in the Experimental Data (6 deg) and for Model 4 (7 deg). Another similarity is that the changes of mind are earlier in the trajectory when commitment is modeled (Fig. 5), as also occurs in the experimental situation. This happens because the later trajectories are more likely to be close to a target, which with commitment makes it more difficult to have a change of mind. Again, this finding can be quantified with the average trajectories, with now the final approach to target across the diagonal for the Experimental Data (final angle from vertical: 55 deg) and Model 4 (angle: 55 deg) but almost horizontal for Model 3 (angle: 90 deg). In general, we expect that the effect of the embodied motor feedback upon the trajectories may depend on task characteristics such as the amount of sensory uncertainty [44]. Thus there may be some experimental situations with trajectories that better resemble Model 4 (as here) and others more like Model 3, depending upon the relative contribution, or gain, of the motor feedback and sensory information to the decision making process.

**Fig 6:**
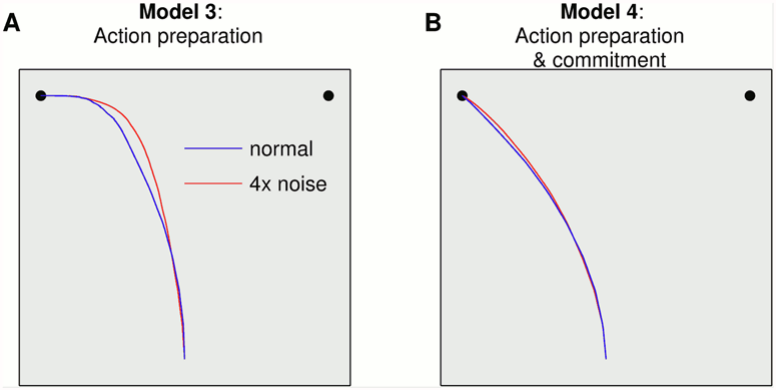
Trajectory dependence on noise. The ‘normal’ average trajectories are taken from Fig. 5. A second average trajectory was computed with increased noise *σ* = 4 (red curve).

This dependance on the experimental situation is also evident from considering that the ‘curvature’ of mouse movements is greater for more difficult choices, even when there are no changes of mind. Fig. 6 shows the average trajectories produced by the Models 3 and 4 for the stimuli used so far and with stimuli having four-fold higher (4×) levels of noise (*i.e.* a more difficult choice). These two conditions closely resemble the comparison between high- and low-frequency words in [16] and the comparison between low- and highly-ambiguous stick figures in [42]. In Model 3, the curvature of mouse movements (as measured for example by its ‘area under the curve’ [17]) is greater in more difficult choices, as is consistent with the empirical result that, even without changes of mind, higher levels of uncertainty ‘curve’ the mouse trajectories. Meanwhile, in Model 4 with commitment, the curvature is still affected but to a lesser degree; this gives an experimental prediction that with commitment, the trajectory will be less sensitive to changes in stimulus noise. The trajectories of both Model 3 and Model 4 are compatible with the results of an experiment that explicitly manipulated decision uncertainty. The study compared two choice conditions having different levels of certainty (100% vs. 75%) and found a small but significant difference in mouse movement curvature [45]. The currently available data cannot clearly adjudicate between the models. We expect these similarities and differences can be controlled to quantify further with specific experimental procedures for examining commitment, such as by manipulating task urgency [46].

### Study 2: Decision speed and accuracy from embodied choice

#### Baseline model: drift-diffusion model with instantaneous action

**Fig 7:**
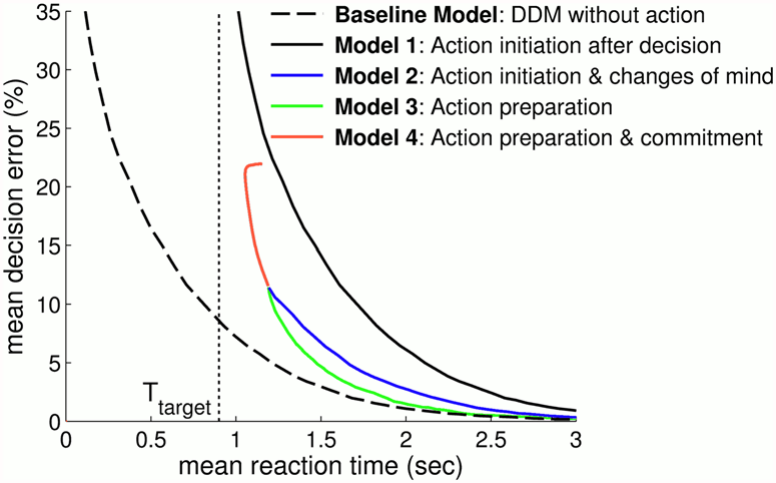
Comparison of 4 decision making with action models and a Baseline Model without action. Each model is characterized by a speed-accuracy curve obtained from simulating (100000 samples) over a range of decision boundaries, with mean decision error plotted against mean response time. The minimal time-to-target *T*_target_ for action completion is also shown (dotted line). Note that curves for Model 3 (green line) and Model 4 (red line) do not overlap, but span different response times.

In the Baseline Model, we consider just the drift-diffusion model with an action made instantaneously when the decision variable *z*(*t*) passes a decision boundary. The speed-accuracy trade-off for this model is represented by a plot of mean response time against the mean error rate while varying the decision boundary (Fig. 7, dashed black line). Because no action is necessary to make a decision, unlike the following models, this baseline represents the best possible speed-accuracy trade-off with this simulated sensory information; all of the other considered models will have speed-accuracy trade-offs above baseline (*i.e.* worse mean errors for a given mean RT), with closeness to the baseline indicating their overall performance.

#### Model 1

Because both targets are equidistant from the start location, the effect of action initiation after decision completion is to increase the response times by a constant delay *T*_target_ (time to reach a target; here 0.9 sec). Therefore, the speed-accuracy trade-off curve for this model of ‘action initiation after decision completion’ has the same shape as the baseline drift-diffusion model, but shifted to longer response times (Fig. 7, black curve). Clearly, no decisions can be made quicker than the time-to-target *T*_target_, which now defines a lower bound that the speed-accuracy curve approaches with increasing mean decision error. Because mean errors decrease with increasing mean response time, the rightwards shift of Model 1 compared with the Baseline Model implies its speed-accuracy curve always lies above baseline.

#### Model 2

Simulation results for this model of ‘action initiation and changes of mind’ (Fig. 7; blue plot) reveal a better speed-accuracy trade-off than that of Model 1 (action initiation after decision; no changes of mind) but poorer than the (unattainable) Baseline Model of instantaneous action to target. The better performance with changes of mind indicates that the improvements in decision accuracy compensate for the resulting slower response times in the speed-accuracy tradeoff, consistent with the extra information during making an action now being used rather than ignored. A second implication of permitting changes of mind is that the least mean response time for Model 2 (∼ 1.2 sec) is farther above the time-to-target *T*_target_ than Model 1; in accordance, the maximal mean errors in Model 2 (∼ 12%) are smaller than those permitted in Model 1. These constraints can actually be a disadvantage in some ecological scenarios, because it is not possible to sacrifice decision accuracy to achieve faster response times in contexts where response speed is critical.

#### Model 3

Simulation results for this model of ‘action preparation’ (Fig. 7; green plot) show that the speed-accuracy trade-off for Model 3 resembles but performs better than that for Model 2 (action initiation and changes of mind), and is only slightly poorer than that of the Baseline Model (instantaneous action). The resemblance to the performance of Model 2 is not unexpected, because the above model of action preparation naturally allows changes of mind when the focus point moves from one target to nearby the other. The performance benefits over Model 2 are expected from the response time savings of the preparatory motor response. Note that the minimum possible response time (0.9 sec) is attainable only by making random choices and heading directly to a target; such random choices are not possible in Model 3, and consequently the response times has a minimum bound (1.2 sec) and minimum accuracy (12%). Thus, similarly to Model 2, the present model of action preparation also suffers from not being able to sacrifice accuracy for faster response times, which could be crucial in situations relying on response speed.

#### Model 4

Simulation results for this model of action preparation and commitment (Fig. 7; red plot) show that the speed-accuracy trade-off is again between the Baseline Model (instantaneous selection) and Model 1 (action initiation after decision), like Models 2 and 3 (changes of mind and preparation). However, the commitment shifts the mean response time range to faster than that of Models 2 and 3 (∼ 1–1.2 sec) at the expense of poorer accuracy (∼ 12–25%); in particular, the speed-accuracy curve for Model 4 looks like continuation of Model 3 to quicker response times. Hence, commitment solves the problem that changes of mind (in preparation or otherwise) lead to relatively slow but accurate decision making, whereas some ecological contexts may demand faster response times. The commitment enforces a quicker (but more inaccurate) decision than would be possible otherwise by heading towards the final target earlier in the decision process, but consequently disregarding the later information. Thus, with commitment the accuracy is sacrificed to achieve greater decision speed, appropriate to situations where time is of the essence.

#### Theoretical support for embodied models

Our results show that the serial decision-then-action system has the poorest speed-accuracy trade-off of all considered models, being the slowest to reach decisions for comparable accuracy. This speaks against its application to ecological scenarios where taking action rapidly can be crucial. Parallel decision-and-action models permit faster responses than the serial model for the same accuracy. Our first parallel model (Model 2), similar to the one suggested by [23], permits the revision of initial decisions as changes of mind. A second parallel model (Model 3) includes an action preparation mechanism that permits action initiation before the accumulated information passes a decision bound. This latter model is more accurate than the first parallel model for comparable response times, suggesting an advantage of acting upon partial information. Still, parallel models do not include mechanisms for changing the ongoing decision based on the current action, and thus do not assign action a role in influencing decisions. Embodied choice models, here exemplified by Model 4, include both action preparation and a feedback loop from action to decision systems that, while allowing changes of mind, also produce a commitment effect: changes of mind become less likely as the action nears completion. A principal advantage of commitment is that decision accuracy can then be traded for speed, allowing quicker response times than possible in parallel or serial models. Embodied choice could thus yield particular advantage in ecological situations where decision speed is at a premium.

Our approach here was to compare modifications of the drift-diffusion model in a simple 2AFC task where an action indicates the choice. Without action, the drift-diffusion model implements optimal decision making, because for discrete time it is formally equivalent to the sequential probability ratio test (SPRT) [5], which minimizes a linear cost function of response time and decision errors averaged over many decision trials [47]. With action, the speed-accuracy trade-off curve of the original drift diffusion model is unattainable, because actions have a cost in delaying response time. Moreover, the minimum time to complete an action gives a lower bound on decision speed that can only be attained with random choice. The optimal speed-accuracy curve for decisions with action would thus extend from 50% accuracy at the minimum action time down towards the curve of the original DDM with increasing decision time. Here we found that a parallel model of action preparation (Model 3) early in the decision process approached the performance of the original DDM for relatively long response times; meanwhile, a feedback loop from action to decision gave a commitment effect necessary to achieve response speeds close to the minimum action time (Model 4). It thus appears that urgent situations are aided by a greater contribution of the motor feedback to the decision process, whereas tasks demanding greater accuracy may demand a greater contribution from the sensory information.

As described earlier in the section on ‘Support for embodied models’, we expect that the effect of the embodied motor feedback on the decision process may be task dependent. The results in this section reinforce that expectation because fast response times were only attained for Model 4, with a large contribution of the motor feedback relative to the sensory information; meanwhile, superior accuracies but with slower response times were attained in Model 3 with no motor feedback but a mechanism for preparation. Taken together, both preparation and commitment within a framework of embodied choice are necessary for covering the full speed-accuracy range.

## Discussion

The embodied choice framework treats action as a proper part of the decision making process rather than a way to report an already made choice (as in serial models) or a component where the accumulated information drives movement during the decision process (as in continuous flow, parallel models). Traditional decision making models were developed mainly to account for laboratory tasks with simplified action choices, which we believe has hindered a full understanding of the importance of action for ecologically relevant decision making. Indeed, living organisms evolved to deal with ecological decisions and the selection of life-or-death actions (such as fight, flight and foraging) rather than laboratory tasks. Given this evolutionary background, it is plausible that decision making and sensorimotor control systems are highly integrated in the brain [48, 49]. Embodied choice is a framework for situated decisions that have similar characteristics to those found in ecologically valid environments, and in doing so makes apparent the importance of action, its dynamics and its constraints within the decision making process.

At the moment, such embodied models are in their infancy. Here we presented a general framework highlighting the importance of action for decision; within this framework, specific models can be designed and tested that include mechanisms such as action preparation and commitment that are currently not considered or considered only partially in current theories of decision-making. Indeed, although we have constructed the present proposal around the drift-diffusion model [1], we expect the overall formalism should be similar for any model based on an evolving decision variable, whether that variable relates to leaky competing accumulators [2], estimated probability [6, 7, 50–52] or an urgency signal [46]. Some empirical support for embodied models already exists but clearly much remain to be done to test current embodied models of choice and develop better ones. The ultimate ambition of this framework is to provide reference models to study ecologically valid choice, in the same way as drift-diffusion and related models have provided (and still provide) a theoretical support to understand perceptual decisions in the laboratory.

Although the results in this paper were characteristic of mouse tracking studies, similar results have been observed also for other behavioral paradigms. In a speeded reaching task where subjects knew the target only probabilistically, their initial movement trajectory approached the mean of the goal selection distribution and then veered toward the goal when revealed [53]; see also [54]. These findings cannot be explained by current parallel models (not even the “changes of mind” model of [23]) because they lack a mechanism for *action preparation* and use the evolving sensory representation only for decision-making and not also for movement preparation and planning. Action preparation dynamics could also explain why the biomechanical costs of movement and target distance influence choice, as reported in [29, 30].

Another key component of the embodied choice framework is a *commitment* mechanism that, once an action is initiated towards a decision choice, makes target revision less likely. Such a mechanism might reflect both the situated aspects of the choice (*e.g.* biomechanical constraints) and the cognitive cost of changes of mind. A recent neurophysiological study of a two-choice reaching task reported evidence of a commitment effect at both behavioral and neuronal levels, with suppression of M1 activity tuned to the unselected target [38]. Other studies that did not directly test the commitment hypothesis reported evidence that is congruent with this idea. For example, it has been consistently reported that early evidence can have greater influence on the final perceptual choice: a primacy effect that can be explained within the leaky competing accumulator framework by adding an *inhibition dominance* mechanism [55] or in terms of *hysteresis* mechanisms in dynamical systems models [3]. Yet this same evidence could, in principle, also support the commitment hypothesis discussed here. That being said, the two phenomena are different: inhibition dominance and hysteresis depend on processes that are internal to the decision system (*e.g.* attractor dynamics in neuronal populations), whereas commitment would be due to the embodied and situated nature of the choice. In situated cognition theories, the current movement trajectory can be considered an *external memory* of the ongoing decision that both biases and facilitates the underlying choice computations by *offloading* them onto the environment [56]. Overall, the exact relations between inhibition dominance and hysteresis (due to neuronal dynamics) versus commitment (due to the embodiment of choice) remain to be studied, and experiments that distinguish between them are required. For example, a possible way to test the commitment hypothesis is to influence the initial stages of action movements without directly changing the evidence accumulation process, such as by studying whether (irrelevant) visuomotor priming can bias a perceptual decision.

Several aspects of the relation between decision and action remain to be investigated. For the sake of simplicity, we reduced the complexity of action dynamics (assuming, for example, constant velocity). A more realistic model of action and its biomechanics is required to fully formalize a fully embodied model, by directly considering action costs (see [32]). For example, the present embodied choice models have action initiation at the start of the trial, whereas relevant data (*e.g.* [29]) show a short deliberation period. Quantifying in more detail the action dynamics and the costs of action in the model of embodied choice should in principle allow the movement initiation to depend on the evolving decision, because a movement that is initiated too prematurely might be too costly to reverse afterwards. A comprehensive formalization should also consider the situated characteristics of the task, such as the fact that different tasks can differ in their intrinsic geometries for movement. For example, if two targets are at different distances, the trajectory that makes them equiprobable is not straight but a curve ending at their center-point.

More generally, the embodied choice framework suggests that choices are not insensitive to the experimental paradigm used to study them. Different experimental set-ups with different action constraints (*e.g.* buttons at various distances; the presence or absence of a deadline and its length; differing kinematic costs for making a movement; using a keyboard, mousetracker, tablet, *etc*.) can induce different choices or even different choice strategies with the same sensory information. For example, the framework predicts that in set-ups with buttons at different distance, the more uncertain choices should be biased towards the closest button, which requires less biomechanical costs at least in conditions of high uncertainty. It also predicts that once initiated an action should be harder to revise in the light of new evidence as an effect of commitment. Recent studies provide preliminary empirical support for these predictions (see [57–59]) although much remains to be investigated. Furthermore, experiments with different deadlines and time constraints will change the way actions are executed; for example, an empirical prediction of embodied choice that remains to be tested is that in urgent situations trajectories tend to be straighter. These considerations do not imply that decision making is a volatile phenomenon or cannot be studied empirically, but rather that its full understanding at the conceptual, computational and neuronal level cannot omit the action dynamics.

Another important direction of research is to link the embodied choice framework with state-of-the-art formal theories of action control, such as optimal control [60], and so permit modeling choice as the *simultaneous optimization* of decision and action. This view is shared by recent theories suggesting that motor control is essentially a decision making process that can be modeled with Bayesian decision theory [61, 62]. However, the proposal made here takes a complementary approach in suggesting that action optimization is also part of the decision making. In this way, it should balance the benefits of making the correct decision (*e.g.* clicking the proper button) with its costs (time and biomechanical constraints to reach it). In general, it is an open question how to attain decision optimality if action is required to make a choice—or whether our brains do actually optimize the choice process or just use heuristics that are ‘good enough’. Whatever the underlying mechanism, our results here indicate that elements of embodied choice should be included to successfully model decisions in ecologically-relevant scenarios.

The neuronal structures that could support embodied models of choice are incompletely known. The proposal made here is largely consistent with recent decision making theories that recognize the close connections between decision and action systems. For example, according to the *intentional framework*, choice recruits sensorimotor circuits that are ultimately responsible for initiating actions or action plans [35]. Along similar lines, perceptual decision making has also been proposed to be *action selection* [63]. Another recent proposal is that choice is implemented as a *distributed consensus* between multiple brain circuits calculating outcome-related and action-related aspects of a decision [64]. These ideas are convergent and largely complementary to the proposed embodied choice framework and might offer testable proposals on the putative neuronal substrate underlying embodied choice. For example, in this article, we have suggested that commitment effects could be implemented by biasing the fixation point to be closer to the currently closest target, but the neuronal underpinnings of this mechanism remains unspecified. One possibility is that such biases are directly incorporated in a ‘biased competition’ process between choice alternatives, as also suggested by the *distributed consensus* model [64]. Another candidate mechanism is the use of corollary discharge and/or the action trajectory as evidence for decision making (in addition to sensory evidence). These hypotheses remain for empirical study.

To conclude, the embodied choice framework has wider scope than a theory of decision making. Indeed, it is a clear example that embodied phenomena (here action movements) can have causal influence on so-called central cognition (here perceptual choice). We make the case that as cognitive processes are studied in ever more ecological contexts, the importance of the cognitive embodiment will become increasingly apparent [65–67].

## Models

This section describes the mathematical and computational details of the models of decision making that were used to generate the results in this paper. All models are built on the drift-diffusion model of Ratcliff and colleagues [1, 40]. A decision variable *z*(*t*) represents the sensory information accumulated to time *t* from unbiased starting information *z*(0) = 0. For convenience, we discretize time in uniform steps Δ*t*, so that the update equation is

$$\begin{array}{c} z\left(t+\Delta t\right)=z\left(t\right)+\Delta z,\;\Delta z∼N\left(\mu ,\sigma^{2}\right), \end{array}$$ (7)

where Δz is the increment of sensory information received at time *t*, which is conventionally assumed drawn from a (stationary) normal distribution *N*(*μ*, *σ* ^2^) of mean *μ* and variance *σ* ^2^. Here we assume *μ* = 1/3 and *σ* = 1, with step-size Δ*t*=50 ms. We then employ various criteria to make decisions from this accumulated sensory information.

(i) *Baseline Model: Drift-diffusion model without action*: The decision criterion in the standard drift-diffusion model is that the accumulated sensory information crosses one of two decision boundaries, assumed equal and opposite at *z* = ±*b*. Here we consider values of *b* between 0.01 and 10 (considered in 31 increments of boundary value). We consider crossing the boundary +*b* to represent a correct decision of error zero and crossing the boundary −*b* an incorrect decision of error one. Results of the model simulations are averaged over 10000 runs, to decrease the variance in estimating the mean decision error and mean response time for each boundary value.

(ii) *Model 1: Action initiation after decision completion*: We now require that an action must be made to one of two equidistant targets to indicate the choice. For simplicity, we assume a basic action model that moves a point (*x*, *y*) along a trajectory with constant speed *v* = 2 units/sec, in a two-dimensional arena from starting point (0, 0) with targets 1,2 at positions (∓1, 1.5). In this first model, we assume that the movement is initiated when the decision variable passes a decision boundary and the trajectory is a straight line to the target. This action model can be represented by having an action focus that is coincident on the chosen target for times *t* ≥ *t*_dec_, as represented in equation (3). Prior to the decision time, there is no choice and the agent cannot move (equivalently, the focus is at the start location). Then the effect of action initiation after decision completion is to increase decision times by a constant delay $T_{\text{target}}=\sqrt{1^{2}+1.5^{2}}/2=0.9$ sec, the time to reach the target.

(ii) *Model 2: Action initiation and changes of mind*: We now consider a decision model in which an action must be made to indicate the choice of a target, as in Model 1, but augmented with a mechanism for ‘changes of mind’ during the movement to the target [23]. This is implemented by updating the decision variable with new sensory information during the movement, with the alternative target selected if the accumulated information *z*(*t*) passes the opposing decision barrier before the trajectory reaches a target. This action model can be represented by having an action focus that is coincident on the currently chosen target, as represented in equation (4). Similarly to Model 1, if the accumulated information has not passed threshold, then there is no choice and the agent cannot move.

(iii) *Model 3: Action preparation*: Next, we consider a model of preparatory motor response from the start of the decision process, rather than waiting to reach a decision boundary to initiate action [24]. Here we consider a preparatory move towards a focus point between the two targets, to approach the most likely target prior to accumulating sufficient information to make a decision. Mathematically, this focus point is defined as collinear between the two targets with distance from each in the proportion ∣*b* + *z*∣ : ∣*b* − *z*∣ for −*b* ≤ *z* ≤ +*b*, and at target 1 for *z* ≤ −*b* or target 2 for *z* ≥ *b*. Mathematically, we define the focus as collinear with the two targets with distance from each in the proportion ∣*b* + *z*∣ : ∣*b* − *z*∣ for −*b* ≤ *z* ≤ *b*, as represented in equation (5). This range is bounded such that the focus is coincident with a target if the decision bound is passed. Hence, the decision bound no longer defines decision termination directly, but rather the embodied choice follows indirectly from action completion (upon reaching a target).

(iv) *Model 4: Action preparation and commitment*: Our final model considers both a preparatory motor response, as in Model 3, and commitment to an action when sufficiently engaged in moving towards a target. Mathematically, we implement commitment by including an additional position-dependent term *g*(*d*_1_ − *d*_2_)/(*d*_1_ + *d*_2_) in the update equation (7), with *d*_1_, *d*_2_ the distances to the two targets, as represented by equation (6) and *g* a gain for the degree of commitment (here set to *g* = 4*b*). All other details are unchanged from Model 3.
